# Using the unmet obstetric needs indicator to map inequities in life-saving obstetric interventions at the local health care system in Kenya

**DOI:** 10.1186/s12939-014-0112-4

**Published:** 2014-12-12

**Authors:** Elizabeth Echoka, Dominique Dubourg, Anselimo Makokha, Yeri Kombe, Øystein Evjen Olsen, Moses Mwangi, Bjorg Evjen-Olsen, Jens Byskov

**Affiliations:** Centre for Public Health Research Institute, Kenya Medical Research Institute (KEMRI), P.O. Box 20752-00202, Nairobi, Kenya; Woman and Child Health Research Center, Department of Public Health, Institute of Tropical Medicine Nationalestraat 155, 2000, Antwerpen, Belgium; Department of Food Science, Jomo Kenyatta University of Agriculture and Technology, PO Box 62000-00200, Nairobi, Kenya; Centre for International Health, University of Bergen, P.O. Box 7804, 5020 Bergen, Norway; Stavanger University Hospital, P.O Box 8100, 4068 Stavanger, Norway; Department of Obstetrics and Gynaecology, Sørlandet Hospital, Flekkefjord, Norway; Centre for Health Research and Development, Faculty of Health and Medical Sciences, University of Copenhagen, Thorvaldsensvej 57, Frederiksberg, DK 1871 Denmark

**Keywords:** Kenya, Life-saving, Pregnancy, Unmet Obstetric Needs, Emergency Obstetric Care

## Abstract

**Background:**

Developing countries with high maternal mortality need to invest in indicators that not only provide information about how many women are dying, but also where, and what can be done to prevent these deaths. The unmet Obstetric Needs (UONs) concept provides this information. This concept was applied at district level in Kenya to assess how many women had UONs and where the women with unmet needs were located.

**Methods:**

A facility based retrospective study was conducted in 2010 in Malindi District, Kenya. Data on pregnant women who underwent a major obstetric intervention (MOI) or died in facilities that provide comprehensive Emergency Obstetric Care (EmOC) services in 2008 and 2009 were collected. The difference between the number of women who experienced life threatening obstetric complications and those who received care was quantified. The main outcome measures in the study were the magnitude of UONs and their geographical distribution.

**Results:**

566 women in 2008 and 724 in 2009 underwent MOI. Of these, 185 (32.7%) in 2008 and 204 (28.1%) in 2009 were for Absolute Maternal Indications (AMI). The most common MOI was caesarean section (90%), commonly indicated by Cephalopelvic Disproportion (CPD)–narrow pelvis (27.6% in 2008; 26.1% in 2009). Based on a reference rate of 1.4%, the overall MOI for AMI rate was 1.25% in 2008 and 1.3% in 2009. In absolute terms, 22 (11%) women in 2008 and 12 (6%) in 2009, who required a life saving intervention failed to get it. Deficits in terms of unmet needs were identified in rural areas only while urban areas had rates higher than the reference rate (0.8% vs. 2.2% in 2008; 0.8% vs. 2.1% in 2009).

**Conclusions:**

The findings, if used as a proxy to maternal mortality, suggest that rural women face higher risks of dying during pregnancy and childbirth. This indicates the need to improve priority setting towards ensuring equity in access to life saving interventions for pregnant women in underserved areas.

## Introduction

Maternal mortality ratio (MMR) that is, number of maternal deaths per 100,000 live births, is a mandatory indicator for measuring progress towards Millennium Development Goal (MDG) five in many countries. Recent global estimates indicate that MMR was highest in sub-Saharan Africa (640), followed by South Asia (280), Oceania (230) and South-Eastern Asia (160) [1]. These estimates underscore the consensus that maternal mortality remains a major challenge to health systems around the globe [1,2]. There is evidence, however, that this burden could be reduced if all women had access to life saving obstetric interventions [3-12], even in low income countries. A systematic review of trends in MMRs showed huge disparities even among countries with similar low economic status. As examples, Lesotho and Ivory Coast, with Gross National Income per capita of 1,000 US$ recorded an increase in MMR from 590/100,000 to 964 - 994/100,000 between 1980 and 2008. Over the same period, Bangladesh with even less income (520 US$) experienced a decrease in MMR from 1329/100,000 live births to as low as 338/100,000 live births [13].

In Kenya, the demographic and health survey of 2009 showed a MMR of 488/100,000 [14]. This was an increase from 414/100,000 in 2003 [15]. Kenya was among eleven countries that contributed to 65 percent of all maternal deaths in 2008 on a global scale. Kenya is also among 23 countries in sub-Saharan Africa making no progress towards achieving the target of MDG five [1]. One year remains to the deadline to reduce maternal mortality from 414/100,000 live births in 2003 to 147/100,000 by 2015 in Kenya [16]. Achievement of this target presents a key challenge and is unlikely to be realised [17].

While the MMR is a useful indication of maternal health service effectiveness, it is argued that it is a poor guide to policy. The indicator does not provide information about what interventions are needed and where [18]. The MMR may reflect how bad a situation is, or what it is correlated with, but does not indicate what to do, where and for whom [19]. In addition, the indicator is based on national estimates [12,20], therefore less operational at sub-national levels of the health system. At the district level, for instance, measuring MMR as a routine may be a challenge. This is because the district health services do not often have the resources and capacity to collect the data required [21], besides the large sample size required for valid measurements [22]. This presents lack of insights on the magnitude of maternal health burden, on its localisation and on possible solutions. Thus, decision-makers in the local health care system may not know how and where to intervene.

The acknowledgement of challenges in measuring the MMR [22-24] led to development of indicators that measure improvements in access and use of services most likely to reduce maternal mortality [8,12,20,25-30]. A set of emergency obstetric care (EmOC) process indicators therefore exist [12,20]. The indicators are based on the understanding that to reduce maternal deaths, certain types of obstetric services must be available and used by women during pregnancy, labour and postpartum [7-9,12,20]. Reductions in maternal mortality and morbidity will therefore depend on countries’ capacity to identify and improve these services [31].

Appreciation of the need to measure access to services likely to reduce maternal mortality has further led to the development of the unmet obstetric needs (UON) indicator [32]. This alternative process indicator concerns maternal mortality. It is based on evidence that in any population, a proportion of pregnant women (1-2%) will develop life-threatening obstetric conditions during pregnancy and childbirth [33-37]. If they receive rapid medical care, nearly all will survive. If they fail to receive appropriate assistance, it will most likely result in maternal deaths [38-40].

**Figure 1:**
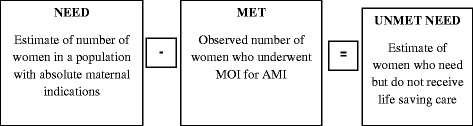
**Operational expression of the unmet obstetric needs concept.**

Briefly, UON refers to the difference between what the health care system should provide to deal with obstetric problems in a given population and the care it actually provides [32]. Figure 1 shows the operational expression of the UON concept.

The UON indicator provides knowledge of the nature and magnitude of the need for essential obstetric care in a defined geographical area [32]. The indicator is therefore appropriate for identifying geographical differences in access to life saving obstetric interventions [12,41]. It further provides answers as to whether pregnant women are receiving the major obstetric interventions they need, where those with unmet needs are, and how many they are. The MMR does not address any of these.

It is suggested that the UON was created in and to be used in low income countries [32,41]. Indeed, UON has been applied in several African countries to measure deficits in obstetric care, contributing to changes in maternal health practice in some. In Koutiala Mali, UON findings showed that more than 100 women in need of obstetric care never reached the hospital and probably died as a consequence. This not only created awareness of service deficits, but also triggered operational measures to tackle the problem, including considerations for coverage and quality of obstetric care [37]. In Tanga region, Tanzania, it was demonstrated that UON data could be used to plan and monitor trends in responsiveness of the health system at intervals. Since the data were generated by stakeholders in the system, they shared the optimism that the findings could revitalise discussions on access to obstetric care both at local and national level [34]. In Mtwara, Tanzania, geographical mapping of UON was useful in priority setting as the findings highlighted deficiencies in the provision of maternity care [35]. In Taounate, Morocco, UON findings created awareness among health personnel on the magnitude of a previously ignored problem, leading the team to identify technical and systemic solutions to address the problem [21].

This paper is based on application of the UON concept to assess the magnitude and distribution of unmet obstetric needs at district level in Kenya. Data presented is part of the “response to accountable priority setting for trust in health systems” (REACT) study 2006-2011, that introduced an intervention to improve equity and access to quality health care at district level in Kenya, Tanzania and Zambia. EmOC was one of the service areas selected to assess whether improved fairness and legitimacy in priority setting processes could have an influence on service output and outcome after an active promotion of fairness conditions. The REACT study focus on quality, equity and trust is closely associated with the specific UON indicator in terms of its importance to avert maternal morbidity and mortality. More details of the REACT study are found elsewhere [42]. Findings presented in this paper provide insights on the importance of additional process indicators that can map inequities in life-saving obstetric interventions at the local health care system in high maternal mortality settings.

## Methods

### Study design and setting

This was a facility based retrospective survey conducted in Malindi District, Kenya (currently Malindi and Magarini sub counties in Kilifi County) in the year 2010. The area is located in the Northern Coastal region, covering an area of 7, 792 square kilometres. Four divisions, namely, Malindi, Langobaya, Marafa and Magarini constitute the district. The total population in the district was 400,514 people in 2009, with a distribution of 140, 739 people in urban and 259, 775 in rural areas [43]. Malindi Division has a higher population density than the other three divisions as it has favourable topographic features and economic factors affecting human settlement. Malindi Town, the main urban centre in the district, is located in Malindi Division. The district had a total of 105 public and private health facilities [44]. Of these, 42 (40%) offer delivery services. One public and two private hospitals provide caesarean section services. The total fertility rate in the district was 4.8 children per woman of reproductive age and a crude birth rate of 38.1/1000 [45].

### Data collection

Identification and listing of all comprehensive EmOC (perform caesarean section and blood transfusion) facilities in the district were undertaken prior to data collection. All divisions, locations and sub locations were also identified and coded.

The UON indicator restricts its scope to a standard list of Absolute Maternal Indications (AMI), that is, maternal life threatening conditions for which major obstetric surgery is performed to solve the problem [32]. The list of AMIs is based on the degree of severity of the indication, the relative stability of its incidence and relatively reproducible diagnosis [19,33]. The standard list of AMI adopted for this study included:

- Antepartum haemorrhage (placenta praevia or abruptio placenta);
- Abnormal presentations (transverse lie or shoulder presentation, face with persistent mento-posterior position or brow presentation);
- Major CPD (mechanical CPD, small pelvis including pre-rupture and rupture of uterus);
- Uncontrollable postpartum haemorrhage.

The list of MOI included:

- Caesarean section;
- Hysterectomy;
- Laporatomy due to uterine rupture.

Based on findings on existence and functionality of EmOC in the district [46], three comprehensive EmOC facilities met the inclusion criteria for the UON study. These were the Government District Hospital and two private hospitals. A nurse-midwife, qualified in assessing obstetric diagnosis, was trained in data collection. A form was filled for every woman who underwent a major surgical obstetric intervention or died in the health facilities in the target district. The possibility of women from the study district having received MOI in other districts was taken into account by reviewing records from the regional referral hospital to identify if the hospital had received any cases from the target district. The referral hospital was the Coast Province General Hospital, located 160 kilometers away from the study area, in Mombasa town.

Data were collected on major obstetric interventions, the maternal indications, geographical origins of the women, and outcomes for mothers. The data were collected retrospectively for the periods 1^st^ January 2008 to 31^st^ December 2009. The principal data source was the operating theatre registers, where most MOI were recorded. Information about the indications for the interventions and other personal data on the women was obtained from patient delivery files, maternity ward registers and admission records for maternity or surgical wards. In filling the unmet obstetric need form, particular attention was paid to the way in which diagnoses were formulated in the registers, and recorded as closely as possible to the way they were usually expressed in clinical language. Where more than one indication for an intervention was performed, all were recorded.

Validity of unmet obstetric need data was addressed in a number of ways. First, information on the surgical procedures performed for the women were obtained from delivery records, theatre registry and patients’ personal files to maximize comprehensiveness and consistency. Incomplete case records were cross matched with information from the three sources to provide a consistent determination of indications and outcomes. Calculation and analysis of the UONs were done for a given population (in a defined geographical area). The place of origin of the patient was therefore specified in the questionnaire. To get a more comprehensive picture and trends in unmet obstetric needs in the district, data for 2008 and 2009 were collected and analysed separately.

### Data analysis

The unmet obstetric need indicator was determined using the following formula:

**Unmet Obstetric Needs** = (EB × RR) − (MOI for AMI)

Where:

**EB** = Expected births in the population: Obtained by multiplying the number of persons in a defined area, during a specified period by the crude birth rate for that region (expected births = population × crude birth rate).

**RR** = Reference rate (1.4%): The low-end estimate of the proportion of deliveries that require a MOI to avoid a maternal death (95% CI, 1.27% -1.52%). This benchmark has been derived from previous UON studies [33]. The benchmark may be applied to data from more remote or dispersed populations in which women experiencing life-threatening indications die outside the formal health care system [33,38].

**(EB × RR)** = Estimated number of women experiencing absolute maternal indications in the population.

**(MOI for AMI)** = Number of women in the population receiving major obstetric interventions (MOI) for absolute maternal indications (AMI) carried out in the same population during the same period.

Thus, the expected births (EB) for Malindi District were obtained by multiplying the population in 2008 (385,460 persons) and 2009 (400, 514 persons), by the Crude Birth Rate (CBR) for the Coastal region (38.1/1000). The expected MOI for AMI were obtained by multiplying the Expected Birth (EB) by the Reference Rate (RR).

The UON deficits were calculated according to the four divisions in the district and by rural and urban residence. For this study, a woman’s inclusion in an urban or rural area was based on the distance between her residence and the comprehensive care facility. Urban residents were defined as women residing within a radius of 10 kilometres from the comprehensive EmOC facility. Rural residents were defined as women residing more than 10 kilometres from the comprehensive EmOC facility. The UON rates among urban and rural women were compared using Chi-square test of association. The strength of the association was estimated using odds ratios (OR), with corresponding 95% confidence interval. The OR were calculated using the actual number of women who received intervention after developing a complication (actual MOI for AMI), while the denominator was the expected births for the respective year.

Approval to conduct the study was obtained from the Kenya Medical Research Institute’s Ethical Review Committee (Scientific Steering Committee Number. 1808). Written permission was obtained from the Medical Officer of Health in the district prior to visiting the health facilities. All data have been maintained confidential and no individuals will be identified in dissemination of findings.

## Results

### Obstetric interventions performed in 2008 and 2009 in Malindi District

Table 1 shows the distribution of obstetric interventions performed by type of facility. All the three hospitals were located in the urban area. The government hospital performed a majority of the obstetric interventions in both 2008 and 2009.

**Table 1:** **Distribution of women who underwent a major obstetric intervention**

	**2008**	**2009**
**Facility**	**Number (%)**	**Number (%)**
Public hospital	535 (94.5%)	652 (90%)
Private for profit hospital 1	25 (4.4%)	69 (9.5%)
Private for profit hospital 2	6 (1.1%)	3 (0.4%)
Total	**566**	**724**

Table 2 shows that the majority of women were residents of Malindi Division in both 2008 and 2009. Similarly, a majority of women were urban residents.

**Table 2:** **Distribution of women who underwent obstetric interventions by division**

	**2008**	**2009**
**Division**	**Number (%)**	**Number (%)**
Malindi	437 (77.5%)	573 (79.1%)
Magarini	94 (16.6%)	107 (14.8%)
Langobaya	19 (3.4%)	24 (3.3%)
Marafa	14 (2.5%)	20 (2.8%)
**Total**	**566**	**724**
**Area**		
Urban*	345 (61%)	455 (62.8%)
Rural**	221 (39%)	269 (37.2%)
**Total**	**566**	**724**

*Urban area = ≤10 km from EmOC facility; **Rural area = >10 km from the EmOC facility.

### Type of maternal indications in 2008 and 2009

Table 3 shows the distribution of maternal indication in 2008 and 2009. Of the 566 and 724 maternal indications in 2008 and 2009, approximately a third were AMIs. The non-absolute indications comprised hypertensive disorders in pregnancy, foetal indications, controllable postpartum haemorrhage and postpartum sepsis.

**Table 3:** **Distribution of type of maternal indications**

	**2008**	**2009**
**Maternal indication**	**Number (%)**	**Number (%)**
Absolute	185 (32.7%)	203 (28%)
Non-Absolute	381 (67.3%)	521 (72%)
**Total**	**566**	**724**

### Distribution of AMI and MOI in 2008 and 2009

Table 4 shows the most common AMI was CPD (narrow pelvis) in 2008 and 2009 respectively, followed by CPD (not specified) in 2008. Shoulder and brow presentations were the least common AMIs. Caesarean section comprised over 90% of MOI in both 2008 and 2009.

**Table 4:** **Distribution of types of absolute maternal indications**

	**2008**	**2009**
**AMI**	**Number (%)**	**Number (%)**
Uterine rupture	11 (5.9%)	5 (2.5%)
Uterine pre-rupture	6 (3.2%)	7 (3.4%)
Transverse lie	14 (7.6%)	13 (6.4%)
Brow	1 (0.5%)	0
Shoulder	0	1 (0.5%)
Face presentation	10 (5.4%)	9 (4.4%)
CPD (macrosomia)	6 (3.2%)	17 (8.4%)
CPD (narrow pelvis)	51 (27.6%)	53 (26.1%)
CPD (not specified)	44 (23.8%)	32 (15.8%)
APH placenta previa	11 (5.9%)	13 (6.4%)
APH abruption placenta	31 (16.8%)	52 (25.6%)
**Total**	**185**	**203**

### Magnitude and distribution of unmet obstetric needs in Malindi District

The findings on unmet obstetric needs are presented as rates of MOI for AMI per 100 expected births and in absolute numbers. Table 5 shows that the overall rate of MOI for AMI per 100 expected births was 1.25% in 2008 and 1.3% in 2009. Compared to the reference rate of 1.4%, it meant that there were unmet needs. In absolute terms, 22 (11%) women in 2008 and 12 (6%) in 2009 who were expected to benefit from an intervention did not.

**Table 5:** **Distribution of unmet needs by divisions**

**2008**	**Expected births**	Expected MOI/AMI 1.4%*	**MOI/AMI performed**	**Absolute deficits**	MOI/AMI rate**
Malindi	7534	105	130	-25***	1.7
Magarini	3578	50	42	8	1.2
Langobaya	1272	18	6	12	0.5
Marafa	2302	32	6	26	0.3
**Total**	**14,686**	**206**	**184**	**22**	**1.25**
**2009**					
Malindi	8272	116	158	-42***	1.9
Magarini	3691	52	28	24	0.8
Langobaya	1228	17	8	9	0.7
Marafa	2068	29	8	21	0.4
**Total**	**15,259**	**214**	**202**	**12**	**1.3**

*Reference rate.

**Actual MOI/AMI for expected births.

***Negative deficits, that is, the number of MOI for AMI performed exceed expected.

Table 5 further indicates that Malindi Division, with a MOI for AMI rate of 1.7% had no unmet need. The negative deficits implied that the number of MOI for AMI performed was exceeded. The other three divisions had MOI for AMI rate of less than 1.4%, implying that women from these areas had unmet needs.

Table 6 shows the MOI for AMI rates between the rural and urban residence in the district. The urban areas had no deficits. The odds ratios (OR) suggest that a pregnant woman with an AMI in the urban area was 3 times more likely to get a life-saving intervention compared to a rural woman.

**Table 6:** **Distribution of MOI for AMI rate by rural-urban residence**

**2008**	**Expected births**	Expected MOI/AMI 1.4%*	**MOI/AMI performed**	**Absolute deficits**	MOI/AMI rate**	**OR (95% CI); p-value**
Urban	4875	68	108	-40***	2.2	2.9 (2.14–3.94)
Rural	9811	138	76	62	0.8	Reference
**Total**	**14,686**	**206**	**184**	**22**	**1.25**	
**2009**						
Urban	5470	76	119	-43***	2.1	2.6 (1.94–3.48)
Rural	9789	138	83	55	0.8	Reference
**Total**	**15,259**	**214**	**202**	**12**	**1.3**	

*Benchmark or low end estimate.

**Actual MOI/AMI for expected births.

***Negative deficits, i.e. number of MOI for AMI performed exceed expected.

**Figure 2:**
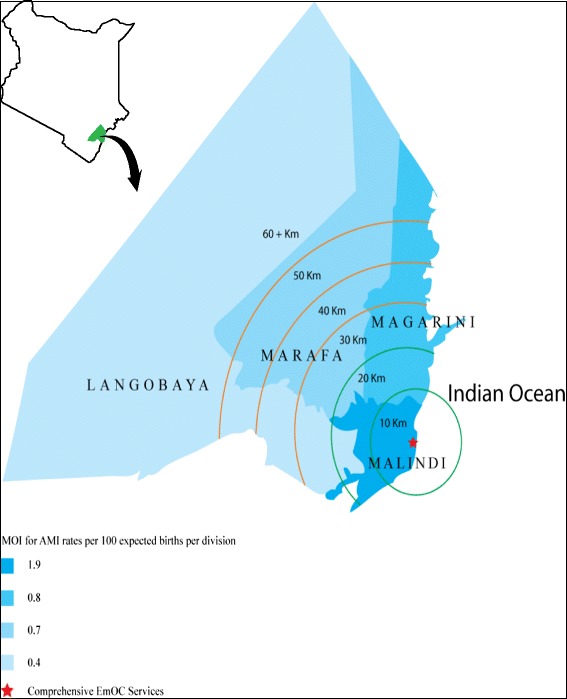
**Distribution of MOI for AMI rates per 100 expected births in 2009 per in division in Malindi District, Kenya.**

Figure 2 shows the variations in magnitude and distribution of UONs between the four divisions in relation to access to the main comprehensive care facility in the district. A notable decrease in MOI for AMI rates with distances from the comprehensive care facilities was observed. Two divisions, Marafa and Langobaya, which were not served by any EmOC facility had rates lower than the reference rate. This implied that women from these divisions had unmet needs. The divisions were not connected to the major trunk road with regular transport to the comprehensive EmOC hospitals. In contrast, Malindi and Magarini Divisions, which were connected to the major trunk road, had no unmet needs.

## Discussion

This paper documents the application of the UON concept to assess the magnitude and distribution of unmet obstetric needs at district level in Kenya. This is the first study to apply the UON concept in Kenya. The findings showed that the rate of MOI for AMI were 1.25% in 2008 and 1.3% in 2009. These are below the reference rate of 1.4% used in this study. This indicated that there were unmet obstetric needs. To appreciate the significance of these figures, the deficits in absolute terms meant that 22 (11%) out of 206 women in 2008 who were expected to benefit from an intervention did not get it. In 2009 12 (6%) out of 214 women who were expected to benefit from an intervention failed to get it.

### Methodological issues and study limitations

Some limitations as well as methodological issues in the application of the UON concept in this study were observed and call for some caution when applying the UON concept. The UON indicator restricts its scope to maternal life-threatening conditions for which a major obstetric surgery is performed to solve the problem [32,41]. It is thus more demanding in terms of data quality. The first limitation in this study was related to the retrospective rather than prospective nature of data collection. Analysis for UON rests on the comparison of MOI rates according to origin of the woman. Therefore, effort must be devoted to making sure that the origins of the women are correct both in the numerator (notification of origins in the sources used in the collection of data) and the denominator (urban and rural population of reference). The retrospective nature of the study denied the investigators a chance to probe the women or their relatives regarding the “right address” upon admission. The possibility of some women on admission to hospital giving a temporary address (near hospital) instead of the real address of residence could not be ruled out. Such situation could give rise to overstatement of the number of such women (who gave a temporary address “in town” instead of their real address), thus the negative deficits observed in the urban area. The issue of bypassing is a common finding in other UON studies, suggesting that women travel from rural areas to deliver in facilities located in urban areas [47,48].

The issue of negative deficits is acknowledged as a major limitation in other UON case studies [33]. There are suggestions that the bias could be minimized in prospective UON studies, where more attention is devoted to probing to ascertain origin of the women. In routine use of UON at district level, this limitation could be mitigated by putting in place measures at facility level to improve reliability of data from records. In Mali for example, daily cross-checking of data entered in both the maternity and operating theatre registers, assigning a surgeon the responsibility of notifying a reliable diagnosis in the operating theatre register, and having a new register for referred cases from health centres and for evacuations to the regional hospital were some of the measures put in place to improve UON data quality. In return, routine use of the UON indicator not only had the effect of triggering consideration of coverage, but also of quality of obstetric care [37].

The second limitation in this study was related to the reference rate used to calculate the expected MOI for AMI rates. Choice of the reference rate is very crucial in a UON study [38]. Ideally, a local reference derived from data in areas close to a care hospital is recommended. However, in this study, due to the issue of bypassing as earlier highlighted, it was not feasible to calculate a local reference rate. Similar limitations are noted in other UON studies [33,35]. Nevertheless, the mapping of deficits in terms of the health system capacity to treat obstetric complications as instituted in this study provided some evidence for creating awareness and initiating dialogue with decision makers on the need to mobilise resources to improve coverage in obstetric care in the underserved areas of Malindi District.

### Magnitude and distribution of unmet needs

A well-functioning district health care system should be capable of providing medical care to all [49]. In this study, although there was an adequate coverage of comprehensive EmOC services in the district as shown in a previous study [46], geographical inequities to this care for rural women were revealed. In obstetrics, a maternal death provides evidence of unmet need [38]. It therefore means that a woman who does not receive appropriate care in the event of a life-threatening obstetric complication may likely die. In addition, since coverage in obstetric “need” is restricted to interventions carried out in order to save a mother’s life, the unmet need can be a proxy to maternal mortality [22,38,41]. The findings in this study may therefore suggest that whose needs were unmet probably died. The observed differences in the deficits between the urban and rural areas further suggest that the rural women had a higher risk of dying or suffering an obstetric disability. Differences in maternal outcomes, such as maternal mortality between urban and rural areas are reported elsewhere [50-52]. Similar patterns showing variations in MOI for AMI rates between rural and urban areas are documented from other UON studies carried out in Benin, Burkina Faso, Mali and Niger [33] and Tanzania [34,35]. Distance to facilities certainly explained the variations in unmet obstetric needs within divisions and also because the comprehensive EmOC facilities were located in the urban areas. In Ethiopia, rural urban inequities in utilisation of EmOC services were partly explained by the care hospital being located in the urban setting [53]. Similar trends, showing fewer interventions with increase in distance from comprehensive care facilities are documented in other UON studies [33-35]. For example, the average distance that women had to travel to reach a hospital varied from 43 in Burkina Faso to 103 kilometres in Niger [33].

The UON concept provided the difference between the number of women who needed life-saving obstetric surgery and the number of women who received such care in Malindi District. The findings therefore provided a measure of the district capacity to treat obstetric complications. The UON indicator also identified geographical areas where unmet needs were largest. In the large REACT study [42], EmOC was one of the service areas that assessed the extent in which fairness and legitimacy were guiding priority setting in health care and whether their strengthening could have an influence on service output and outcome. Findings from this paper show that priority setting seemed insufficient to address obstetric needs in the district. This observation is documented in other REACT study findings [54].

## Conclusions

The findings presented in this paper provide insights on importance of additional process indicators that can map inequities in life saving obstetric interventions at the local health system.

The UONs in rural areas in this study indicate that access to life saving obstetric care is a challenge for rural women. If used as proxies to maternal mortality, the findings suggest that rural women in the district face very high risks of dying during pregnancy and childbirth. The findings support the need identified by others as well, to improve priority setting processes to better influence decision making on how to achieve optimal coverage and access to life-saving obstetric services for pregnant women in the district. Improving coverage entails providing decision makers with knowledge on the extent of need and how to intervene.

## Abbreviations

AMI: Absolute Maternal Indication
APH: Antepartum haemorrhage
CPD: Cephalopelvic Disproportion
EmOC: Emergency Obstetric Care
EB: Expected births
MMR: Maternal mortality ratio
MOI: Major obstetric intervention
UON: Unmet Obstetric Needs

## References

[1] WHO, UNICEF, UNFPA, World Bank (2010). Trends of maternal mortality: 1990 to 2008 estimates developed by WHO, UNICEF, UNFPA and The World Bank.

[2] Hogan MC, Foreman KJ, Naghavi M, Ahn SY, Wang M, Makela SM, Lopez AD, Lozano R, Murray CJL (2010). Maternal mortality for 181 countries, 1980—2008: a systematic analysis of progress towards Millennium Development Goal 5. Lancet.

[3] Prual A, Bouvier-Colle MH, De Bernis L, Breart G (2000). Severe maternal morbidity from direct obstetric causes in West Africa: incidence and case fatality rates. Bull World Health Organ.

[4] Ronsmans C (2001). How can we monitor progress towards improved maternal health goals?. SHSOP.

[5] Koblinsky MA (2003). Reducing Maternal Mortality. Learning from Bolivia, China, Egypt, Honduras, Indonesia, Jamaica, and Zimbabwe.

[6] Wagstaff A, Claeson M (2004). The millennium development goal for health. Rising to the challenges.

[7] Paxton A, Maine D, Freedman L, Fry D, Lobis S (2005). The evidence for emergency obstetric care. Int J Gynecol Obstet.

[8] WHO: World Health Report 2005 (2005). Make Every Mother and Child Count.

[9] Campbell OM, Graham WJ (2006). Strategies for reducing maternal mortality: Getting on with what works. Lancet.

[10] Freedman LP, Graham WJ, Brazier E, Smith JM, Ensor T, Fauveau V, Themmen E, Currie S, Agarwal K (2007). Practical lessons from global safe motherhood initiatives: Time for a new focus on implementation. Lancet.

[11] Lawn JE, Lee AC, Kinney M, Sibley L, Carlo WA, Vinod K, Pattinson R, Darmstadt GL (2009). Two million intrapartum-related stillbirths and neonatal deaths: where, why, and what can be done?. Int J Gynecol Obstet.

[12] WHO, UNFPA, UNICEF and AMDD (2009). Monitoring emergency obstetric care, a handbook.

[13] Nyamtema AS, Urassa DP, Van Roosmalen J (2011). Maternal health interventions in resource limited countries: a systematic review of packages, impacts and factors for change. BMC Pregnancy Childbirth.

[14] Kenya National Bureau of Statistics and ICF Macro (2010). Kenya Demographic and Health Survey 2008-09.

[15] Central Bureau of Statistics, Ministry of Health and ORC Macro (2004). Kenya Demographic and Health Survey 2003.

[16] Ministry of Public Health and Sanitation and Ministry of Medical Services (2009). National Reproductive Health Strategy: 2009-2015.

[17] RoK and UNDP (2010). Draft Progress in Attainment of MDGs and Way Forward Towards Achieving MDGs by 2015 in Kenya.

[18] Bailey P, Paxton A, Lobis S, Fry D (2006). Measuring progress towards the MDG for maternal health: including a measure of the health systems capacity to treat obstetric complications. Int J Gynecol Obstet.

[19] De Brouwere V, Laabid A, Van Lerberghe W, Mundigo A, Obermeyer C, An alternative to the maternal mortality ratio (1996). The coverage of obstetric interventions need. Seminar on Innovative Approaches to the Assessment of Reproductive Health.

[20] WHO, UNICEF, UNFPA (1997). Guidelines for monitoring the availability and use of obstetric services.

[21] Belghiti V, De Brouwere V, Kegels G, Van Lerberghe W (1998). Monitoring unmet obstetric need at district level in Morocco. Trop Med Int Health.

[22] Graham WJ, Ahmed S, Stanton C, Abouzahr CL, Campbell OM (2008). Measuring maternal mortality: an overview of opportunities and options for developing countries. BMC Mede.

[23] Abouzahr C, Wardlaw T (2001). Maternal mortality at the end of a decade: signs of progress?. Bull World Health Organ.

[24] Yazbeck AS (2007). Challenges in measuring maternal mortality. Lancet.

[25] Ronsmans C, Achadi E, Sutratikto G, Zazri A, McDermott J (1999). Use of hospital data for safe motherhood programmes in south Kalimantan, Indonesia. Trop Med Int Health.

[26] Liljestrand J (1999). Reducing perinatal and maternal mortality in the world: the major challenges. BJOG.

[27] Donnay F (2000). Maternal survival in developing countries: what has been done, what can be achieved in the next decade?. Int J Gynecol Obstet.

[28] Goodburn E, Campbell OM (2001). Reducing mortality in the developing world: sector-wide approaches may be the key. Brit Med J.

[29] Ronsmans C, Oona MRC, Jeanne MD, Koblinsky M (2002). Questioning the indicators of need for obstetric care. Bull World Health Organ.

[30] Gabrysch S, Zanger P, Seneviratne HR, Mbewe R, Campbell OM (2011). Tracking progress towards safe motherhood: meeting the benchmark yet missing the goal? An appeal for better use of health-system output indicators with evidence from Zambia and Sri Lanka. Tro Med Int Health.

[31] Paxton A, Bailey P, Lobis S, Fry D (2006). Global patterns in availability of emergency obstetric care. Int J Gynecol Obstet.

[32] Unmet Obstetric Needs Network. (1999). Tackling Unmet Obstetric Needs. Part 1: Concepts, General Principles and International Network. [http://www.uonn.org/pdf/Guide1.pdf] accessed December 2014

[33] Unmet Obstetric Needs Network (2002). L’approche des BesoinsObstétricaux Non Couverts pourles Interventions ObstétricalesMajeuresEtude comparativeBénin, Burkina-Faso, Haïti,Mali, Maroc, Niger, Pakistan et Tanzanie.

[34] Prytherch H, Massawe S, Kuelker R, Hunger C, Mtatifikolo F, Jahn A (2007). The unmet need for Emergency Obstetric Care in Tanga Region, Tanzania. BMC Pregnancy Childbirth.

[35] Hunger C, Ku¨lker R, Kitundu H, Massawe S, Jahn A (2007). Assessing unmet obstetric need in Mtwara Region, Tanzania. Trop Med Int Health.

[36] De Groof D, Harouna Y, Bossyns P (2003). Application of the Unmet Obstetrical Needs method in the III neighbourhood of Niamey, Niger (1999)]. Bull Soc Pathol Exot.

[37] Guindo G, Dubourg D, Marchal B, Blaise P, De Brouwere V (2004). Measuring unmet obstetric need at district level: how an epidemiological tool can affect health service organization and delivery. Health Pol Plann.

[38] De Brouwere V, Laabid A, Van Lerberghe W (1996). Evaluation des besoins en interventions obstétricales au Maroc; une approche fondée sur l’analyse spatiale des déficits.[Estimating need for obstrtrical interventions in Morroco. An approach based on the spatial analysis of deficits]. Rev Epidemiol Sante Publique.

[39] De Brouwere V, Van Lerberghe W (1998). Les besoins obstétricaux non couverts.

[40] De Brouwere V, Tonglet R, Van Leiberghe W (1998). Strategies for reducing maternal mortality in developing countries: What can we learn from history of western countries?. Trop Med Int Health.

[41] Ronsmans C, Brouwere V, Dubourg D, Dieltiens G (2004). Measuring the need for life-saving obstetric surgery in developing countries. BJOG.

[42] Byskov J, Bloch P, Blystad A, Hurtig AK, Fylkesnes K, Kamuzora P, Kombe Y, Kvale G, Marchal B, Martin DK, Michelo C, Ndawi B, Ngulube TJ, Nyamongo I, Olsen OE, Shayo EH, Silwamba G, Songstad NG, Tuba M (2009). Accountable priority setting for trust in health systems – the need for research into a new approach for strengthening sustainable health action in developing countries. BMC Health Res Policy Syst.

[43] KNBS (2010). Kenya Population and Housing Census 2009.

[44] Kenya Master Health Facility List. [http://www.ehealth.or.ke/facilities/latestfacilities.aspx] Accessed July 2012

[45] Macro International Inc: Measure DHS, Stat Compiler. [http://www.measuredhs.com]. Accessed March 2013

[46] Echoka E, Kombe Y, Dubourg D, Makokha A, Evjen-Olsen B, Mwangi M, Byskov J, Evjen Olsen Ø, Mutisya R (2013). Existence and functionality of emergency obstetric care services at district level in Kenya: theoretical coverage versus reality. BMC Health Serv Res.

[47] Leonard KL, Mliga GR, Haile Mariam D (2002). Bypassing Health Centres in Tanzania: revealed preferences for quality. J Afr Econ.

[48] Olsen ØE, Ndeki S, Norheim OF (2005). Availability, distribution and use of emergency obstetric care in northern Tanzania. Health Pol Plann.

[49] WHO (2007). Everybody business: Strengthening health systems to improve health outcomes: WHO’s framework for action.

[50] Ronsmans C, Etard JF, Walraven G, Høj L, Dumont A, De Bernis L, Kodio B (2003). Maternal mortality and access to obstetric services in West Africa. Trop Med Int Health.

[51] Ronsmans C, Graham WJ (2006). Maternal Survival 1-Maternal mortality: who, when, where, and why. Lancet.

[52] Liang J, Dai L, Zhu J, Xiaohong L, Zeng W, Wang H, Li Q, Mingrong L, Zhou R, Wang Y (2011). Preventable maternal mortality: Geographic/rural urban differences and associated factors from the population-based maternal mortality surveillance system in China. BMC Public Health.

[53] Wilunda C, Putoto G, Manenti F, Castiglioni M, Azzimonti G, Edessa W, Atzori A, Merialdi M, Betrán AP, Vogel J, Criel B (2013). Measuring equity in utilization of emergency obstetric care at Wolisso Hospital in Oromiya. Ethiopia: a cross sectional study. Int J Equity Health.

[54] Bukachi SA, Onyango-Ouma W, Siso JM, Nyamongo IK, Muai JK, Olsen OE, Byskov J: **Healthcare priority setting in Kenya: a gap analysis applying the accountability for reasonableness framework.** *Int J Health Plann Man,* 2013, In press. 10.1002/hpm.2197 23775594

